# Effects of Synthetic Serum Supplementation in Sperm Preparation Media on Sperm Capacitation and Function Test Results

**DOI:** 10.1155/2016/1027158

**Published:** 2016-06-16

**Authors:** Ying-Fu Shih, Shu-Ling Tzeng, Wen-Jung Chen, Chun-Chia Huang, Hsiu-Hui Chen, Tsung-Hsien Lee, Maw-Sheng Lee

**Affiliations:** ^1^Institute of Medicine, Chung Shan Medical University, Taichung 402, Taiwan; ^2^Department of Obstetrics and Gynecology, Chung Shan Medical University Hospital, Taichung 402, Taiwan; ^3^Department of Urology, Chung Shan Medical University Hospital, Taichung 402, Taiwan; ^4^Division of Infertility Clinic, Lee Women's Hospital, Taichung 406, Taiwan; ^5^Department of Obstetrics and Gynecology, National Taiwan University and National Taiwan University Hospital, Taipei 100, Taiwan

## Abstract

Albumin supplementation of culture media induces sperm capacitation in assisted reproduction technique cycles. Synthetic serum supplementation is clinically used to replace albumin for preventing transmission of infectious agents. However, the effects of synthetic serum supplementation on sperm capacitation have rarely been investigated. Spermatozoa from 30 men with normal basic semen analysis results were collected, divided into five aliquots, and cultured in capacitating conditions in four combinations of two synthetic serum supplements, serum substitute supplement (SSS) and serum protein substitute (SPS), and two fertilization media, Quinns Advantage*™* Fertilization (QF) and human tubular fluid (HTF) media. Reactive oxygen species (ROS) levels in spermatozoa were measured through chemiluminescence. Furthermore, acrosome reaction and western blotting for tyrosine phosphorylation were used to evaluate sperm capacitation. HTF+SSS had significantly higher ROS levels than QF+SPS did (11,725 ± 1,172 versus 6,278 ± 864 relative light units). In addition, the spermatozoa cultured in QF+SPS had lower motility, acrosome reaction rates, and tyrosine phosphorylation levels compared with those cultured in HTF+SSS. In conclusion, the effects of synthetic serum supplementation on sperm capacitation varied according to the combination of media. These differences may lead to variations in spermatozoon ROS levels, thus affecting sperm function test results.

## 1. Introduction

When passing through the cervix, uterus, and oviducts, human spermatozoa undergo a physiological process called capacitation to become capable of fertilizing oocytes [1]. During capacitation, various cellular changes occur, including generation of a limited amount of reactive oxygen species (ROS) and protein phosphorylation at tyrosine residues [2]. After these alterations, spermatozoa undergo acrosome reaction, in which hydrolytic enzymes enabling spermatozoa to fertilize oocytes are released [3, 4].

In clinical practice, basic semen analysis—which focuses on the concentration, motility, and morphology of spermatozoa, according to the World Health Organization (WHO) guidelines [5]—is used to determine the fertilization potential of human spermatozoa. However, the criteria or requirements for spermatozoa differ for natural fertilization, intrauterine insemination (IUI), in vitro fertilization (IVF), and intracytoplasmic sperm injection (ICSI) [6]. For the management of infertile couples without evident female or male factors, IUI is initially considered. If IUI fails more than three times, IVF cycles are recommended for these patients because the accurate fertilization potential of spermatozoa under these conditions is uncertain.

For infertile couples with normozoospermia, failure of IUI necessitates advanced sperm function tests for determining the fertilization potential of spermatozoa. These tests can be used to generate a standard IVF or ICSI treatment plan [7–9]. Most sperm function tests analyze parts of the capacitation process, such as hyperactivation, sperm-zona binding, and acrosome reaction [7]. The hemizona assay and induced acrosome reaction test are valuable predictors of IVF outcome [7, 9, 10].

ROS is a positive trigger for capacitation-related modifications [11–13]. Donà et al. reported that spermatozoon ROS content directly influences the levels and locations of tyrosine phosphorylation and then enables the spermatozoa to undergo acrosome reaction [14]. Nevertheless, spermatozoa are sensitive to oxidative stress because they have a limited amount of antioxidant enzymes, but they have abundant unsaturated fatty acid on their cell membrane as well as abundant DNA, both of which are targets of free radical attack. Oxidative stress-mediated damage to spermatozoa is a major pathology contributing to male infertility [15–17]. High ROS levels in the seminal fluid impair the sperm DNA integrity and thus inhibit spermatozoon function [18]. Furthermore, infertile men have lower nonenzymatic antioxidant activity in the seminal plasma than fertile men do [19, 20].

Before IUI, IVF, or ICSI, spermatozoa are processed through in vitro preparation, which induces certain levels of sperm hyperactivation. Serum albumin and sodium bicarbonate can induce sperm capacitation during in vitro culturing of spermatozoa [1, 13]. In andrology laboratory settings, synthetic serum supplements for fertilization media are used, rather than albumin, to prevent transmission of infectious agents. However, the effects of synthetic serum supplementation on sperm capacitation during the preparation and insemination period have rarely been investigated.

A recent meta-analysis suggested that, compared with overnight coincubation, a short period of spermatozoon and oocyte coincubation provides more satisfactory IVF outcomes [21]. Therefore, the capacitation process in the IVF settings may need to be more effectively completed within a shorter incubation period. This study was specifically focused on the exact status of functions of spermatozoa (e.g., capacitation events and DNA damage) cultured in synthetic serum-supplemented sperm preparation media for infertile couples with normozoospermia.

## 2. Materials and Methods

### 2.1. Patient Selection and Semen Collection

All experimental procedures were approved by the Institutional Review Board of Chung Shan Medical University Hospital, Taichung, Taiwan (CS07162 and CS14066). To prevent the interference of an infertile etiology (e.g., male, tubal, and ovarian factors), only infertile couples with secondary infertility and an unexplained etiology (UI) were recruited in this study. Semen samples were obtained from 30 male partners in the UI couples. Informed consent was obtained from all participating couples from July 2013 to December 2014.

Basic semen analyses were performed according to the fourth edition of the WHO guidelines after 3–5 days of sexual abstinence. All semen samples showed normal results in the basic semen analysis (sperm count > 20 × 10^6^/mL, motility > 50%, and morphology > 14%) and Endtz test (<1.0 × 10^6^/mL).

For liquefaction before analyses, the semen samples were kept at room temperature for an average of 1 h (range: 0.5–1.5 h). One liquefied neat semen aliquot was used for sperm motion analysis and ROS measurement. Each liquefied semen sample from one man was separated into four aliquots and then cultured in the following four semen preparation media: (1) modified human tubal fluid media (HTF) with 5% serum substitute supplement (SSS; Irvine Scientific, Santa Ana, CA, USA) in the control (HTS) group; (2) HTF with 15% Quinns Advantage Serum Protein Substitute (SPS; SAGE In Vitro Fertilization Inc., Trumbull, CT, USA) in the HTP group; (3) Quinns Advantage Fertilization Medium (QF; SAGE In Vitro Fertilization Inc.) with 5% SSS in the QFS group; and (4) QF with 15% SPS in the QFP group. The protein supplements SSS and SPS are used as a replacement for human serum albumin, a recognized sperm capacitating agent.

To determine the effect of antioxidant supplementation in sperm preparation media, we collected semen samples again from these UI couples. Then, all sperm samples were separated into two aliquots and incubated in the two following sperm preparation media: (1) the HTS group and (2) HTS with 5 mM glutathione in the GSH group.

The liquefied semen was prepared through density gradient centrifugation (DGC) at 300 ×g with PureSperm (Nicadon, Gothenburg, Sweden; 90/45%) for 15 min. The sperm motion and ROS levels of the washed spermatozoa were also analyzed. The spermatozoa were incubated in the aforementioned preparation media at 37°C under 5% CO_2_ for 3 h. After the incubation, acrosome reaction rates and tyrosine phosphorylation levels of the spermatozoa were evaluated.

### 2.2. ROS Level Measurements

ROS levels were measured using a chemiluminescence assay with luminol (5-amino-2,3-dihydro-1,4-phthalazinedione; Sigma, St. Louis, MO, USA) as a probe. Samples were prepared as 100 *μ*L aliquots of sperm at 10 × 10^6^/mL with 2.5 *μ*L of luminol, prepared as a 5 mM stock solution in dimethyl sulfoxide (Sigma). Each sample was scanned using a luminometer (FlexStation 3 Benchtop MultiMode Microplate Reader; Molecular Devices, LLC, USA). All samples were measured in duplicate. We then scanned the washed spermatozoon samples for 180 min for detecting the dynamic changes in ROS levels. Here, ROS levels are expressed as relative light units (RLU).

### 2.3. Sperm Motion Analysis

Sperm motion characteristics were analyzed using computer-assisted sperm analysis (CASA; Hamilton Thorne, Inc., Beverly, MA, USA), as per the 1998 guidelines of the European Society of Human Reproduction and Embryology [22]. In brief, the parameters settings for analysis included the following: image acquisition rate 80 Hz; number of spermatozoa sampled ≥200; and number of microscopic fields sampled at 200x magnification ≥1. Chambers used for sperm analysis measured 0.01 mm^2^ in surface area, with a 0.02 mm depth.

CASA was used to determine various sperm parameters, including concentration, motility, average path velocity (VAP), straight line velocity (VSL), straightness of sperm motion (STR), and lateral displacement amplitude of head (ALH). Other measured parameters included the percentage of progressive motile spermatozoa exhibiting a VAP > 25 *μ*m/s and STR > 80%.

### 2.4. Hemizona Assay

After incubation of sperm samples for 2 h, sperm-zona interactions were assessed using our hemizona assay, as described previously [9] but with modifications. In brief, fresh unfertilized oocytes from our assisted reproduction program were used as the source of zona pellucida. After sperm preparation in the various culture media, 20,000 sperm in total were added to a droplet of the media. A pair of hemizona was coincubated at 37°C under 5% CO_2_ in air for 2 h, with spermatozoa either from the QFP group (test) or from the HTS group (control). The number of spermatozoa tightly bound to the zona was counted; the results of the hemizona assay were expressed as the hemizona assay index: the ratio of the number of spermatozoa bound to the test droplet to that of spermatozoa bound to the control droplet.

### 2.5. Acrosome Reaction Evaluation

After incubation of sperm samples for 2 h, the acrosome status was assessed through FITC-PNA staining (Sigma), as described in our previous report [23]. In brief, 20 *μ*L of sperm suspension was spread over a clean microscopy slide, air-dried, fixed in 95% ethanol for 5 min, and again air-dried. The fixed slides were stained using FITC-PNA (600 *μ*L of FITC-PNA in 15.4 *μ*L reagent water in a foil-covered Coplin jar) for 15 min at ambient temperature. The slides were rinsed by dipping them in phosphate-buffered saline (PBS) two times before fixing them for 15 min in paraformaldehyde at ambient temperature. The slides were then air-dried, mounted, and stored in the dark until scoring. Between 100 and 250 spermatozoa were counted per slide and scored. Labeling of only the equatorial segment of the acrosome indicates a normally acrosome-reacted spermatozoon that has lost the outer acrosomal membrane present over the anterior acrosomal cap but has an intact equatorial segment.

### 2.6. Western Blot Analysis of Tyrosine Phosphorylation

After incubation in the capacitating condition, proteins extracted from spermatozoa were analyzed through sodium dodecyl sulphate- (SDS-) polyacrylamide gel electrophoresis (PAGE) and western blot analysis. In brief, samples were resuspended in the Laemmli sample buffer (2% SDS, 10% glycerol, 5% b-mercaptoethanol, and 62.5 mM Tris-HCl, pH 6.8) and heated at 100°C for 5 min. Proteins were then separated through 8% SDS-PAGE and transferred onto a nitrocellulose membrane [24]. Nonspecific binding sites on the membrane were blocked using 5% (w : v) nonfat milk in Tris-buffered saline (20 mM Tris and 137 mM NaCl, pH 7.6). The nitrocellulose membrane (0.22 mm pore size; Micron Separations Inc., Westboro, MA, USA) was incubated overnight at 4°C with an antiphosphotyrosine monoclonal antibody (clone 4G10, 1/1000; Upstate Technology Inc., Lake Placid, NY, USA). The blots were then incubated with a horseradish peroxidase goat antimouse IgG (Kirkegaard and Perry Lab., Gaithersburg, MD, USA) for 1 h. The signals were then detected using an enhanced chemiluminescence (ECL) commercial kit (Amersham Biosciences, Piscataway, NJ, USA), and the relative photographic density was quantified by scanning the photographic negatives on a gel documentation and analysis system (AlphaImager 2000, Alpha Innotech Corporation, San Leandro, CA, USA).

### 2.7. Sperm DNA Damage Assessment

The Calbiochem OxyDNA Kit (Merck KGaA, Darmstadt, Germany), which entails employing an in vitro fluorescent protein binding method, was used to detect oxidative injury to DNA in spermatozoa. One aliquot of a semen sample containing 3 × 10^6^ spermatozoa was pelleted, washed using PBS, and fixed; spermatozoa were permeabilized by incubating them in ice-cold 70% ethanol at −20°C for 1 h. Fixed cells were centrifuged at 1,600 rpm for 5 min, washed with PBS two times, resuspended in 1 mL of wash solution (Tris-buffered saline/Tween 20 containing thimerosal), and pelleted at 1,600 rpm for 5 min. Next, 100 *μ*L of 1x FITC conjugate was added to the cell pellet, which was then incubated in the dark for 60 min at room temperature. The cells were then washed with a wash solution. The fluorescence was read using a flow cytometer at a 495 nm excitation wavelength and 515 nm barrier filter.

DNA fragmentation was evaluated using our terminal deoxynucleotidyl transferase-mediated deoxyuridine triphosphate-biotin nick-end labeling (TUNEL) assay (Boehringer Mannheim, Mannheim, Germany), as reported previously [23]. In brief, the sperm samples were washed in PBS and then centrifuged for collecting spermatozoa at 200 ×g. The spermatozoa were then treated with a solution containing 0.1% Triton X-100 (Sigma). A 30 mL TUNEL mixture was added to the same volume of each sample. The samples were then incubated for 60 min at 37°C in a moist chamber in the dark, washed three times with PBS, and then analyzed through FACS. At least 10,000 cells were counted. The presence of green fluorescent signals indicated positivity.

### 2.8. Statistical Analysis

Sperm motion characteristics, acrosome reaction rates, the intensity of tyrosine phosphorylation on the western blot, oxidative injury rates, and DNA fragmentation rates were subjected to the Wilcoxon signed-rank test to evaluate the differences among the four groups (HTS, HTP, QFS, and QFP) or the difference between two groups (HTS and GSH). A confidence level of *p* < 0.05 was considered the limit of statistical significance.

## 3. Results

**Figure 1:**
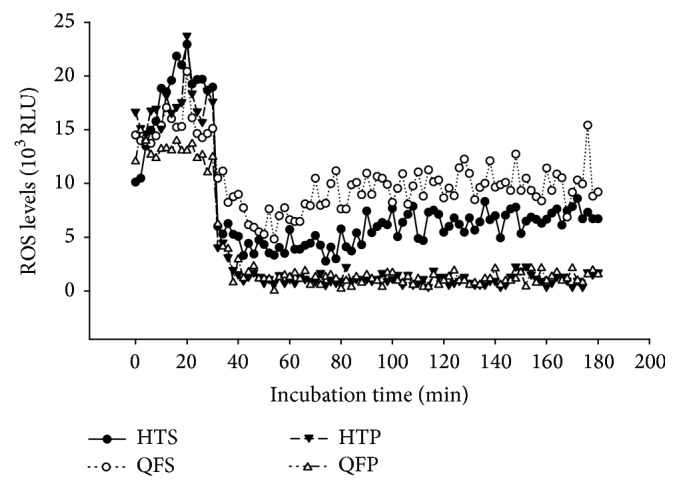
Dynamic patterns of reactive oxygen species (ROS) levels in washed spermatozoa cultured in various sperm preparation media.

We collected 30 semen samples from the male partners of the UI couples in our andrology laboratory. The basic semen analysis results and demographic data of these samples are presented in Table 1. After DGC, each semen sample was divided into four groups according to the sperm preparation media used—QFS, HTS, QFP, and HTP—and cultured for a short incubation period of 2-3 h.  Figure 1 presents the dynamic changes observed in the ROS levels of representative samples during the incubation period. Transient elevation in the ROS levels was noted in all sperm samples. After 3 h of incubation, spermatozoa in the QFS and HTS groups showed consistently higher ROS levels than those in the QFP and HTP groups did.

**Figure 2:**
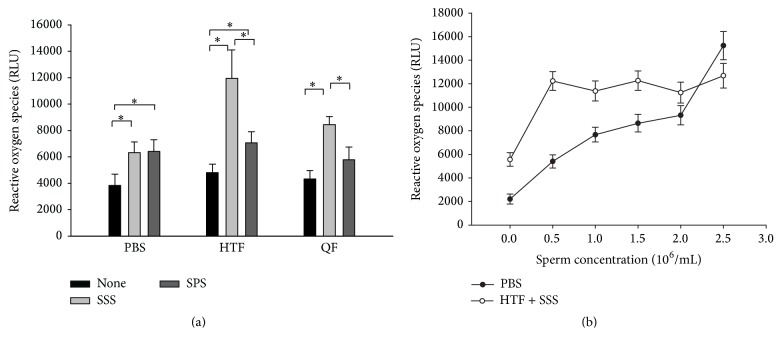
Reactive oxygen species (ROS) levels in the sperm preparation medium components. The data are presented as the mean (SD). RLU denotes relative light units, and *∗* indicates significantly different ROS levels between the two groups according to the Mann-Whitney *U* test.

We selected various combinations of sperm preparation media and serum supplements for ROS measurement and sperm function tests. Sperm preparation media (HTF or QF) with SSS had significantly higher ROS levels than those with SPS did (Figure 2(a)). HTF media with SSS demonstrated the highest ROS levels (11,948 ± 2,162 RLU) after only 2 h of incubation.

For the spermatozoa incubated for 2 h in PBS solution, ROS levels were associated with the sperm concentration (Figure 2(b)). By contrast, the ROS levels were relatively constant for the spermatozoa cultured in HTF+SSS at 0.5–2.5 × 10^6^/mL (Figure 2(b)). All sperm function tests were performed using a sperm concentration of 1 × 10^6^/mL after a 2 h incubation period, unless otherwise specified in Section 2.

We performed two sperm function tests: CASA and the zona binding assay. The motility (median (interquartile range): 65.7% (61.4%–88.8%) versus 51.5% (45.6%–79.7%), *p* = 0.047, by Wilcoxon signed-rank test) and progressive motility (31.2% (27.0%–51.4%) versus 24.3% (15.5%–48.0%), *p* = 0.047, by Wilcoxon signed-rank test) decreased significantly in the QFP group compared with those in the HTS group (Table 2). Similar findings were observed in the zona binding assay. More spermatozoa from the HTS group were bound to the hemizona than those from the QFP group (33.3% (22.7%–46.5%) versus 1%, *p* = 0.021, by Wilcoxon signed-rank test).

**Figure 3:**
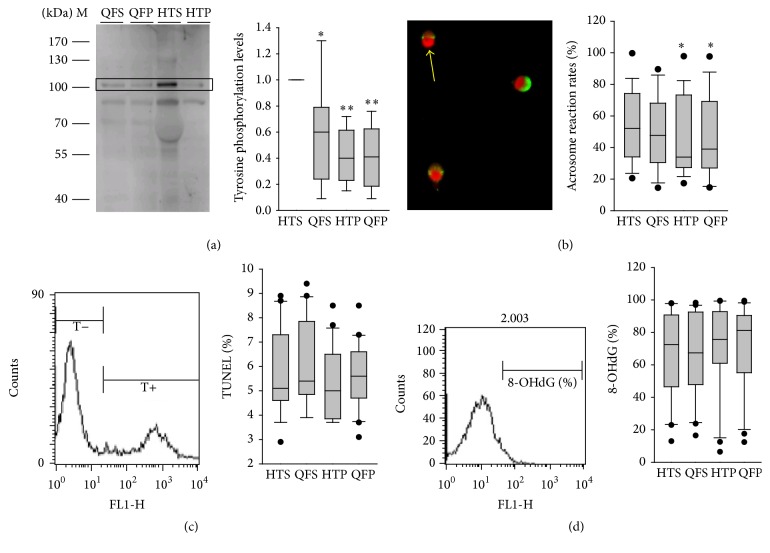
Tyrosine phosphorylation levels of protein of 105 kDa according to western blotting (a), acrosome reaction rates (b), DNA fragmentation according to the TUNEL test (c), and oxidative injury according to the 8-OHdG test (d) in spermatozoa cultured in various sperm preparation media. *∗* and *∗∗* denote *p* < 0.05 and *p* < 0.01, respectively, compared with spermatozoa in the HTS capacitating condition according to the Wilcoxon signed-rank test. The arrow in (b) indicates a spermatozoon with a reacted acrosome. The black circles denote all data points that lie outside the 10th and 90th percentiles.

Acrosome reaction test and western blotting for tyrosine phosphorylation levels (proteins of 105 and 81 kDa) were performed after 2 h of incubation in the four types of media. We demonstrated the protein of 105 kDa as an example. The addition of SPS to QF significantly reduced tyrosine phosphorylation levels (0.46 (0.23–0.67) versus 1, *p* = 0.008; Figure 3(a)) and acrosome reaction rates (39.0% (27.0–69.2) versus 52.1% (34.0–74.2), *p* = 0.024; Figure 3(b)) in the cultured spermatozoa compared with those in the HTS media.

To evaluate the sperm DNA damage caused by ROS or oxidative stress, 8-OHdG and TUNEL assays were performed. Although the ROS levels of spermatozoa cultured in preparation media with SSS supplementation were elevated, the 8-OHdG and TUNEL results did not differ significantly among the spermatozoa cultured in the four preparation media (Figures 3(c) and 3(d)).

**Figure 4:**
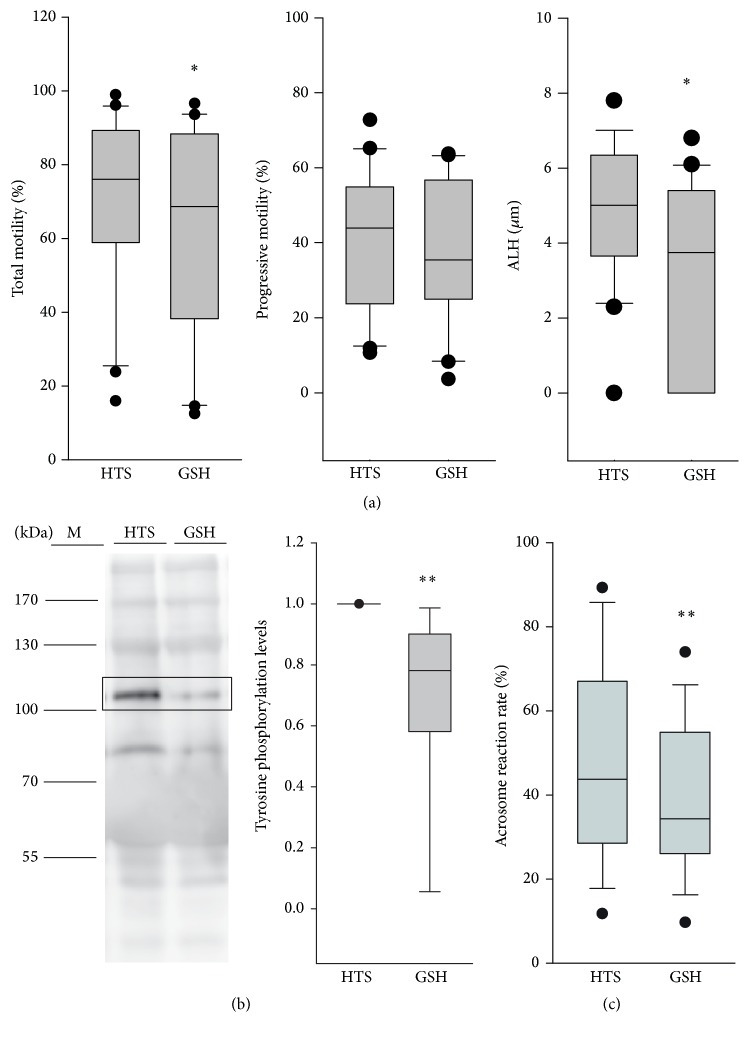
Motion characteristics (a), western blotting for tyrosine phosphorylation levels of protein of 105 kDa (b), and acrosome reaction rates (c) in spermatozoa cultured in various sperm preparation media. *∗* and *∗∗* denote *p* < 0.05 and *p* < 0.01, respectively, compared with spermatozoa in the HTS capacitating condition according to the Wilcoxon signed-rank test. GSH denotes 5 mM glutathione added to the HTS medium.

To test the effect of antioxidants on the capacitation of spermatozoa cultured in the sperm preparation media, 5 mM reduced glutathione was added to the HTS medium (the GSH group). CASA revealed that motility (median (interquartile range): 76.1% (58.9%–89.2%) versus 68.7% (38.2%–88.4%), *p* = 0.014, by Wilcoxon signed-rank test) and ALH (5.0 *μ*M (3.6–6.4 *μ*M) versus 3.8 *μ*M (0–5.4 *μ*M), *p* = 0.018, by Wilcoxon signed-rank test) decreased with the addition of glutathione in the capacitating condition (addition of SSS; Figure 4(a)).

The results of western blotting for tyrosine phosphorylation levels (proteins of 105 and 81 kDa) and immunostaining for acrosome reaction were similar to those of sperm motion analysis. Spermatozoa in the GSH group have significantly decreased tyrosine phosphorylation levels of protein of 105 kDa (0.78 (0.60–0.89) versus 1, *p* = 0.001, by Wilcoxon signed-rank test; Figure 4(b)) and acrosome reaction rates (34.4% (26.5%–54.45) versus 43.7% (29.3%–66.0%), *p* = 0.002, by Wilcoxon signed-rank test; Figure 4(c)) compared with those in the HTS group.

## 4. Discussion

Our results indicated that various commercial synthetic serum supplements could induce sperm capacitation at different levels. The varied sperm capacitation levels and sperm function test results were positively associated with the ROS levels in the preparation media. Furthermore, the addition of glutathione (an antioxidant) reduced the capacitation levels. However, the transient elevations in ROS levels during the sperm preparation process are not directly associated with DNA damage of spermatozoa.

The present study results are consistent with those of previous studies indicating that a limited amount of ROS can trigger the sperm capacitation process [11–13]. When we analyzed each preparation medium component separately, the media supplemented with SSS showed higher ROS levels and a higher proportion of capacitated spermatozoa than those supplemented with SPS did. Taken together, these findings further confirm that some preparation medium components, such as SSS in the present study, can modify the ROS levels of spermatozoa and simultaneously sperm function test results.

Albumin is considered an antioxidant because its molecules contain cysteine-34, which has free sulfhydryl (SH) groups that capture radicals [25]. However, the presence of albumin in culture media facilitates the transfer of free radicals from one molecule to another [26]. Compared with SPS, SSS may have more free SH groups, which facilitate the transfer of ROS during sperm capacitation. This transfer is critical for sperm activation [13].

The strength of the present study is that we used divided spermatozoa from individual patients and cultured them in four sperm preparation media. In theory, they should have demonstrated similar sperm function test results. However, spermatozoa from a single man exhibited significantly different sperm function test results in the various preparation media. The tyrosine phosphorylation levels confirmed that the preparation media, specifically the synthetic serum supplement, induced the varying capabilities of sperm capacitation within a short period (2-3 h) of in vitro incubation. The different sperm function test results induced by the various sperm preparation media may lead to incorrect interpretation regarding the fertilization potential of spermatozoa and consequently overuse or underuse of IVF and ICSI.

The elevated ROS levels in sperm preparation media were not associated with higher oxidative DNA injury or spermatozoon DNA fragmentation. We offer two possible explanations for this observation. First, the elevation of ROS levels was transient (within <30 min) and the increasing DNA injury or fragmentation was not evident in such a short period. A recent study focusing on sperm preparation by using the DGC method for ICSI demonstrated that DNA fragmentation levels decreased after DGC but gradually and nonsignificantly increased during a short incubation period of 2 h [27]. We used DGC as the sperm preparation method, which increases the ROS levels and reduces DNA fragmentation in the spermatozoa [28]. However, the present data indicated that elevated ROS levels do not aggravate DNA fragmentation in a short period of in vitro incubation. Second, our patients featured normal basic semen analysis results. All sperm preparation media could induce substantial capacitation of the spermatozoa, probably sufficient for fertilizing relatively few oocytes. Nevertheless, it remains unknown whether the differences in ROS and capacitation levels after serum supplementation affect the fertilization potential of spermatozoa from patients with inadequate sperm parameters, such as oligozoospermia, asthenozoospermia, and teratozoospermia.

## 5. Conclusion

Serum supplementation of sperm preparation media may alter the ROS levels and modify the function test results of spermatozoa. In IVF settings, the transient elevation in ROS levels does not lead to sperm DNA fragmentation and oxidative injury. However, inaccurate sperm function test results because of elevated ROS levels may lead to overuse or underuse of ICSI. To establish generally applicable criteria for sperm function tests, further investigation is warranted.

## Figures and Tables

**Table 1:** Demographic data and sperm motion characteristics in the semen samples from 30 male partners of couples with unexplained secondary infertility. The data are presented as the median (interquartile range).

Data (*n* = 30)	Median	25%~75% range
Age (years)	33	29~38
Concentration (M/mL)	86.8	49.2~172.9
Morphology (%)	18.3	15~32
Motility (%)	77.3	66.1~83.5
Progressive motility (%)	37.8	28.4~58.7
VAP(*μ*m/s)	28.4	25.5~35.5
VSL (*μ*m/s)	19.5	16.1~22.4
ALH (*μ*m)	1.7	1.0~3.0

**Table 2:** Sperm motion characteristics observed through computer-assisted semen analysis and hemizona assay results of the washed spermatozoa after 2 h of incubation. The data are presented as the median (interquartile range).

	HTS (HTF+SSS)	QFS (QF+SSS)	HTP (HTF+SPS)	QFP (QF+SPS)
Motility (%)	65.7 (61.4~88.8)^a^	60.1 (47.7~94.1)	59.0 (42.6~73.1)	51.5 (45.6~79.7)^a^
Progressive motility (%)	31.2 (27.0~51.4)^b^	28.2 (22.2~54.4)	27.4 (21.3~37.6)	24.3 (15.5~48.0)^b^
VAP(*μ*m/s)	29.0 (27.0~33.6)	39.8 (29.5~46.6)	27.9 (32.4~38.4)	29.8 (27.0~37.0)
VSL (*μ*m/s)	15.4 (13.4~17.0)	20.6 (16.7~27.3)	16.6 (15.7~21.6)	14.8 (12.1~18.3)
ALH (*μ*m)	4.2 (3.2~5.2)	4.6 (1.7~6.5)	5.0 (2.9~5.6)	4.6 (0~5.8)
Hemizona assay (%)	100	—	—	33.3 (22.7~46.5)^c^

^a^ *p* = 0.047, ^b^ *p* = 0.047, and ^c^ *p* = 0.021 by Wilcoxon signed-rank test.

## References

[1] De Jonge C. (2005). Biological basis for human capacitation. *Human Reproduction Update*.

[2] Liguori L., de Lamirande E., Minelli A., Gagnon C. (2005). Various protein kinases regulate human sperm acrosome reaction and the associated phosphorylation of Tyr residues and of the Thr-Glu-Tyr motif. *Molecular Human Reproduction*.

[3] O'Flaherty C., Beorlegui N., Beconi M. T. (2003). Participation of superoxide anion in the capacitation of cryopreserved bovine sperm. *International Journal of Andrology*.

[4] Olds-Clarke P. (2003). Unresolved issues in mammalian fertilization. *International Review of Cytology*.

[5] World Health Organization (1999). *WHO Laboratory Manual for the Examination of Human Semen and Sperm-Cervical Mucus Interaction*.

[6] Weber R. F. A., Dohle G. R., Romijn J. C. (2005). Clinical laboratory evaluation of male subfertility. *Advances in Clinical Chemistry*.

[7] Oehninger S., Franken D. R., Sayed E., Barroso G., Kolm P. (2000). Sperm function assays and their predictive value for fertilization outcome in IVF therapy: a meta-analysis. *Human Reproduction Update*.

[8] Liu D. Y., Baker H. W. G. (2003). Disordered zona pellucida-induced acrosome reaction and failure of in vitro fertilization in patients with unexplained infertility. *Fertility and Sterility*.

[9] Lee T.-H., Liu C.-H., Huang C.-C., Chen H.-H., Chen S.-U., Lee M.-S. (2008). The association between polypronucleate zygote formation with certain motion characteristics of sperm and IVF outcome. *Journal of Assisted Reproduction and Genetics*.

[10] Oehninger S., Franken D. R., Ombelet W. (2014). Sperm functional tests. *Fertility and Sterility*.

[11] Aitken R. J., Harkiss D., Knox W., Paterson M., Irvine D. S. (1998). A novel signal transduction cascade in capacitating human spermatozoa characterised by a redox-regulated, cAMP-mediated induction of tyrosine phosphorylation. *Journal of Cell Science*.

[12] O'Flaherty C., de Lamirande E., Gagnon C. (2005). Reactive oxygen species and protein kinases modulate the level of phospho-MEK-like proteins during human sperm capacitation. *Biology of Reproduction*.

[13] de Lamirande E., Lamothe G. (2009). Reactive oxygen-induced reactive oxygen formation during human sperm capacitation. *Free Radical Biology and Medicine*.

[14] Donà G., Fiore C., Tibaldi E. (2011). Endogenous reactive oxygen species content and modulation of tyrosine phosphorylation during sperm capacitation. *International Journal of Andrology*.

[15] Said T. M., Agarwal A., Sharma R. K., Mascha E., Sikka S. C., Thomas A. J. (2004). Human sperm superoxide anion generation and correlation with semen quality in patients with male infertility. *Fertility and Sterility*.

[16] Agarwal A., Prabakaran S., Allamaneni S. (2006). What an andrologist/urologist should know about free radicals and why. *Urology*.

[17] Tremellen K. (2008). Oxidative stress and male infertility—a clinical perspective. *Human Reproduction Update*.

[18] Moustafa M., Sharma R. K., Thornton J. (2004). Relationship between ROS production, apoptosis and DNA denaturation in spermatozoa from patients examined for infertility. *Human Reproduction*.

[19] Mostafa T., Tawadrous G., Roaia M. M. F., Amer M. K., Kader R. A., Aziz A. (2006). Effect of smoking on seminal plasma ascorbic acid in infertile and fertile males. *Andrologia*.

[20] Song G. J., Norkus E. P., Lewis V. (2006). Relationship between seminal ascorbic acid and sperm DNA integrity in infertile men. *International Journal of Andrology*.

[21] Huang Z., Li J., Wang L., Yan J., Shi Y., Li S. (2013). Brief co-incubation of sperm and oocytes for in vitro fertilization techniques. *The Cochrane Database of Systematic Reviews*.

[22] European Society for Human Reproduction and Embryology (1998). Guidelines on the application of CASA technology in the analysis of spermatozoa. ESHRE Andrology Special Interest Group. *Human Reproduction*.

[23] Lee T.-H., Liu C.-H., Shih Y.-T. (2010). Magnetic-activated cell sorting for sperm preparation reduces spermatozoa with apoptotic markers and improves the acrosome reaction in couples with unexplained infertility. *Human Reproduction*.

[24] Towbin H., Staehelin T., Gordon J. (1979). Electrophoretic transfer of proteins from polyacrylamide gels to nitrocellulose sheets: procedure and some applications. *Proceedings of the National Academy of Sciences of the United States of America*.

[25] Miura T., Muraoka S., Ogiso T. (1992). Oxidative damage to bovine serum albumin induced by hydroxyl radical generating systems of xanthine oxidase + EDTA–Fe^3+^ and ascorbate + EDTA–Fe^3+^. *Chemico-Biological Interactions*.

[26] Otsuki J., Nagai Y., Chiba K. (2009). Damage of embryo development caused by peroxidized mineral oil and its association with albumin in culture. *Fertility and Sterility*.

[27] Rougier N., Uriondo H., Papier S., Checa M. A., Sueldo C., Alvarez Sedó C. (2013). Changes in DNA fragmentation during sperm preparation for intracytoplasmic sperm injection over time. *Fertility and Sterility*.

[28] Aitken R. J., Finnie J. M., Muscio L. (2014). Potential importance of transition metals in the induction of DNA damage by sperm preparation media. *Human Reproduction*.

